# Real-world evidence of lower-dose intensity of immune checkpoint inhibitors in MSI-H/dMMR gastrointestinal cancers from a resource-constrained setting

**DOI:** 10.3389/fonc.2026.1766420

**Published:** 2026-05-04

**Authors:** Mohamad Mourad, Noura Abbas, Maya Charafeddine, Ali Shamseddine

**Affiliations:** Department of Internal Medicine, Hematology/Oncology Division, American University of Beirut Medical Center, Beirut, Lebanon

**Keywords:** conflict-affected area, dMMR (deficient mismatch repair), financial toxicity, gastrointestinal tumor, LMIC (low- and middle-income countries), low dose ICI, MSI-H, resource-constrained settings

## Abstract

**Background:**

Immune checkpoint inhibitors (ICIs) have markedly improved outcomes in metastatic microsatellite instability-high (MSI-H) or deficient mismatch repair (dMMR) gastrointestinal (GI) malignancies. However, in fragile settings, especially in low- and middle-income countries (LMIC) and areas of conflict, access remains severely limited by prohibitive costs and supply chain disruptions. Starting in late 2019, Lebanon’s economic crisis led to health system collapse, currency devaluation, medication shortages, and ongoing conflict, which necessitated adaptive treatment strategies. Early pharmacodynamic studies suggest lower ICI doses may achieve comparable efficacy; however, real-world evidence from conflict-affected settings where dose reductions occur by necessity rather than design remains absent. This study examines ICI dosing outcomes during Lebanon’s acute crisis period.

**Methods:**

We conducted a retrospective analysis of adult patients with metastatic MSI-H/dMMR GI cancers treated with ICIs at the American University of Beirut Medical Center between 2018 and 2024, encompassing Lebanon’s economic collapse and acute health system fragility. Dose-intensity was calculated as the percentage of actual dose received compared to the recommended dose. Patients were stratified into <75% and ≥75% dose-intensity groups based on actual doses delivered under resource constraints. The primary endpoint was progression-free survival (PFS); secondary endpoints included overall response rate (ORR), clinical benefit rate (CBR), and immune-related adverse events (irAEs). Exploratory endpoint was overall survival (OS).

**Results:**

Twenty-nine patients were included, 19/29 patients (65.5%) had colorectal cancer. 18/29 patients (62%) received ≥75% of the recommended dose-intensity, while eleven (38%) received <75%. ORR was 63.6% and 66.7% respectively (p = 0.362), while CBR was 90.9% and 72.2% respectively. No statistically significant difference in PFS or OS was observed. All irAEs (n=4; 14%) occurred in patients receiving ≥75% dose-intensity and included grade 3 diarrhea, grade 3 interstitial nephritis and erythroderma and grade 2 polyarthritis.

**Conclusions:**

In LMIC with a conflict-affected health system and experiencing medication shortages and economic collapse, lower dose-intensity ICIs may be feasible for MSI-H/dMMR GI malignancies, offering a pragmatic approach to maintain treatment continuity when standard dosing is inaccessible. These findings are exploratory, highlighting the need for larger prospective studies and the importance of integrating health system context, resilience, and adaptation into oncology research in fragile settings.

## Introduction

Microsatellite instability-high (MSI-H) or deficient mismatch repair (dMMR) represents a small subset of metastatic gastrointestinal (GI) malignancies. It is more prevalent in early-stage disease than in advanced stages. In the metastatic setting, MSI-H/dMMR tumors are relatively rare compared to microsatellite-stable (MSS) tumors, with reported rates of 2% in pancreatic adenocarcinoma, and approximately 5% in metastatic colorectal and gastric cancers (1–3).

MSI-H/dMMR status leads to disruption in DNA repair mechanisms and accumulation of mutations, resulting in a high neoantigen load and enhanced tumor immunogenicity. For this reason, MSI-H/dMMR tumors are more likely to respond to immune checkpoint inhibitors (ICIs), which restore anti-tumor immune responses by “releasing the brakes” on T-cell activity (4). The introduction of ICIs has transformed the treatment landscape for these malignancies, achieving superior outcomes compared to conventional chemotherapy in both metastatic and non-metastatic settings (2, 5). Not only do MSI-H/dMMR GI malignancies respond better to ICIs, but also emerging evidence suggests that the use of chemotherapy in these cancers might even be detrimental for certain cases. For instance, in resectable MSI-H/dMMR gastric cancer, patients who received chemotherapy had a worse prognosis than those treated with surgery alone (6).

Despite their efficacy, the widespread use of ICIs is restricted by their prohibitive cost. In the United States (US), the median annual cost of immunotherapy is about three times the median annual income, and this disparity is even greater in low- and middle-income countries (LMICs). Therefore, without third-party coverage, patients who rely solely on personal income to cover treatment costs are often unable to afford these medications (7). In Lebanon, a single vial of pembrolizumab (100mg) costs approximately $2,700, and a vial of nivolumab (40mg) costs around $575 (8). With a national gross domestic product (GDP) per capita of $3,654.4 in 2022, the price of one cycle of ICI can exceed the average annual income (9). Furthermore, a recent study in Lebanon that enrolled 4,540 participants found that 60.9% did not have health insurance and 53.7% had a low income, which indicates that the majority of the Lebanese patients may not have access to ICIs if needed (10).

Beginning in late 2019, Lebanon’s economic crisis led to sharp currency devaluation and a profound deterioration of the healthcare system, ultimately causing medication shortages and restricting access to numerous therapies including anti-cancer medications (11, 12). Despite the significant financial burden of cancer care, it is often overlooked in emergency preparedness plans; a problem that is especially pronounced in LMIC where healthcare systems are further strained by geopolitical instability (13). In these settings, providing quality healthcare is often challenging due to disruptions in routine services, growing patient needs, resource constraints, and exposure to multiple public health emergencies (14). In a fragile and conflict-affected country such as Lebanon, with severe resource constraints, and an unstable healthcare system, strategies to reduce treatment costs were essential.

The use of lower doses of ICIs was explored at our institution and was supported by the initial studies of these agents. Initial pharmacodynamic studies of pembrolizumab and nivolumab have demonstrated that PD-1 receptor saturation occurs at very low doses (1mg/kg for pembrolizumab and 0.3mg/kg for nivolumab), and lower doses showed comparable response rates to higher doses across different tumors. Furthermore, in the early phase I and II trials of pembrolizumab, a dose of 2mg/kg was used for non-small cell lung cancer (NSCLC) and melanoma, and despite its proven efficacy, subsequent phase III trials used higher doses (200mg flat dose for NSCLC and 10mg/kg for melanoma), and eventually U.S. Food and Drug Administration (FDA) ultimately approved a flat dose of 200mg (15–22). Due to the financial toxicity of ICIs, currently there are several studies that have explored the use of low doses in various malignancies. Consistent with the initial trials, data from these studies also suggest that lower doses of ICIs achieve comparable clinical outcomes to standard dosing, but at a considerably lower cost (23, 24). A phase III study randomized 500 patients to receive either ultra-low dose of nivolumab (20mg) or chemotherapy in second line treatment for a heterogeneous group of patients with different solid malignancies and 66.2% PD-L1 positivity, demonstrated a median OS of 5.88 months in the nivolumab arm compared to 4.70 months in the chemotherapy arm (P = 0.022 with a hazard ratio of 0.8) with no significant difference in response rates (25).

In this study, we present a retrospective analysis conducted at the American University of Beirut Medical Center (AUBMC), a tertiary care center in Lebanon, between 2018 and 2024, involving patients diagnosed with MSI-H/dMMR GI malignancies, some of whom received lower-than-recommended dose-intensity of ICI due to financial constraints. This study aims to evaluate the outcomes of lower-dose intensity of immunotherapy in a real-world, resource-limited setting.

## Methods

### Study design and participants

This is a retrospective analysis of adult patients diagnosed with metastatic MSI-H/dMMR GI cancers treated with ICIs at the AUBMC between January 2018 and December 2024. MSI-H/dMMR status was defined by the loss of expression of one or more mismatch repair proteins (MLH1, MSH2, MSH6, and PMS2), as determined by immunohistochemistry or by next generation sequencing.

We included adult patients, aged ≥ 18 years, diagnosed with metastatic MSI-H/dMMR originating from any organ of the GI tract, and treated with at least one cycle of ICI therapy at AUBMC during the study period.

### Data collection

Clinical data were extracted from electronic medical records and included demographics (age, gender), tumor characteristics (primary tumor site, KRAS/NRAS/BRAF mutation status), treatment details (line of treatment, type and dosing of ICI, treatment duration), survival outcomes, imaging-based response assessments, and immune-related adverse events (irAEs).

### Treatment exposure and dose-intensity

During the study period, a group of patients received lower-than-recommended dose-intensity of ICIs due to medication shortage and financial constraints. This included either reduced doses per cycle (for pembrolizumab a dose of 2mg/kg or 100mg flat dose was used instead of flat dose of 200mg) and/or extended intervals between treatment cycles which occurred when medications were not available or when patients could not afford treatment.

Dose-intensity was calculated as the actual total dose received during the treatment period divided by the recommended total dose during the same period, expressed as a percentage. The treatment period was defined as the time between the first and last administered ICI cycle. Patients were stratified into two groups based on a dose-intensity cutoff of 75%, which allowed for comparably sized groups for analysis.

### Disease evaluation and response assessment

Baseline disease evaluation was performed using either serial computed tomography (CT) scan of the thorax, abdomen, and pelvis or 18F-labeled fluorodeoxyglucose positron emission tomography-CT (18F-FDG-PET-CT). Imaging was repeated at 3- to 4-month intervals throughout treatment. Treatment response was assessed according to Response Evaluation Criteria in Solid Tumors (RECIST) version 1.1.

### Study endpoints

The primary endpoint of this study was progression-free survival (PFS), defined as the time from initiation of ICI therapy to radiologic or clinical disease progression or death, whichever occurred first, or last follow-up for censored cases.

Secondary endpoints included overall response rate (ORR), defined as the proportion of patients achieving complete or partial response, and clinical benefit rate (CBR) defined as the percentage of patients achieving CR, PR, or stable disease, both as per RECIST version 1.1 criteria, and irAEs, defined as any toxicity attributable to ICI treatment, categorized according to the Common Terminology Criteria for Adverse Events (CTCAE) version 5.0. Exploratory endpoint was overall survival (OS), defined as the time from initial diagnosis to death from any cause or last follow-up for censored cases.

### Statistical analysis

Descriptive statistics were used to summarize baseline characteristics. Continuous variables were reported as medians with ranges, and categorical variables as frequencies and percentages. Patients were stratified into two groups based on ICI dose-intensity: <75% and ≥75%. OS and PFS were assessed using the Kaplan-Meier method, and differences between groups were evaluated with the log-rank test. ORR was compared using Fisher’s exact test. A swimmer’s plot was constructed to illustrate treatment duration, ongoing response, and survival status. A p-value <0.05 was considered statistically significant. All statistical analyses were performed using SPSS version 29.0 (IBM Corp., Armonk, NY).

### Ethical considerations

This study was conducted in accordance with the ethical standards of the Declaration of Helsinki and approved by the Institutional Review Board (IRB) of the AUBMC (IRB ID: BIO-2024-0332). Given the retrospective nature of the study and the use of de-identified patient data, informed consent was waived by the IRB. Strict measures were implemented to ensure patient privacy and confidentiality in accordance with institutional and regulatory guidelines.

## Results

### Patient characteristics

**Figure 1:**
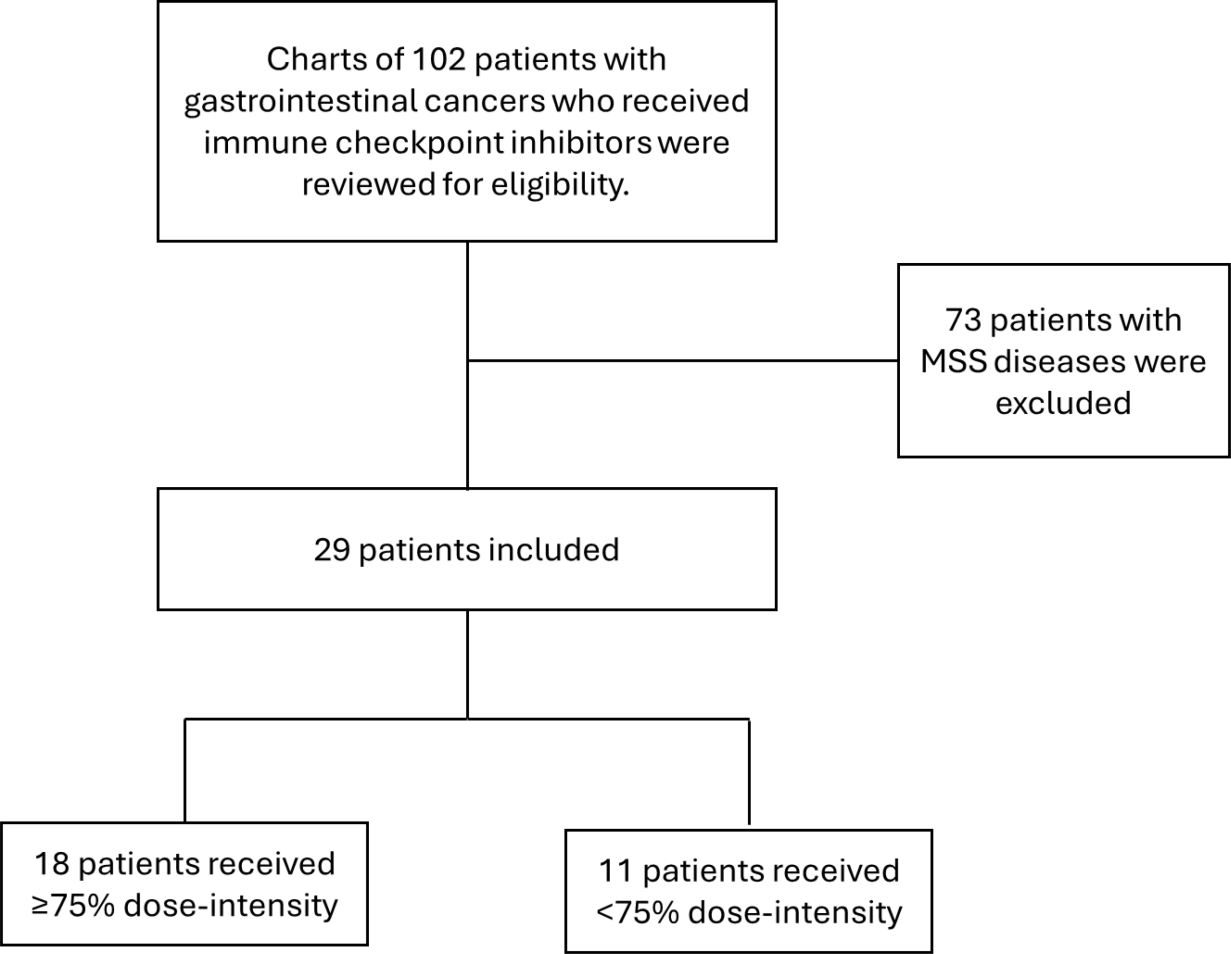
Consort diagram flowchart.

A total of 29 patients with metastatic MSI-H/dMMR GI cancers were identified. The most common diagnosis was colorectal cancer (CRC), accounting for 19/29 patients (65.5%), followed by gastric cancer in 5/29 patients (17.2%). Pembrolizumab was the most frequently used ICI, administered to 24/29 patients (82.8%). 3/29 patients (10.3%) received the combination ipilimumab and nivolumab, 1/29 patient (3.4%) received nivolumab alone, and one patient initially received pembrolizumab but was switched to nivolumab due to medication unavailability. 22/29 patients (72.4%) received ICI as first-line treatment, while 7/29 patients (24.1%) received prior chemotherapy. Based on dose-intensity, 18/29 patients (62%) received ≥75%, while 11/29 (38%) received <75%. Baseline characteristics are summarized in **Table 1**. **Figure 1** shows the flowchart for our cohort.

**Table 1:** Patients’ characteristics.

Characteristic		Overall	Dose < 75% (N = 11)	Dose ≥ 75% (N = 18)
Age, years, median (range)		58 (19-83)	75 (34-83)	51 (19-74)
Gender, n(%)	Male	17 (58.6%)	4 (36.4%)	13 (72.2%)
	Female	12 (41.4%)	7 (63.6%)	5 (27.8%)
Primary tumor site, n(%)	Gastroesophageal junction	1 (3.4%)	0 (0%)	1 (5.6%)
	Stomach	5 (17.2%)	3 (27.3%)	2 (11.1%)
	Gallbladder, liver, bile duct	1 (3.4%)	0 (0%)	1 (5.6%)
	Ampulla	2 (6.9%)	0 (0%)	2 (11.1%)
	Pancreas	1 (3.4%)	0 (0%)	1 (5.6%)
	Small bowel	0 (0%)	0 (0%)	0 (0%)
	Colon	15 (51.7%)	7 (63.6%)	8 (44.4%)
	Rectum	4 (13.8%)	1 (9.1%)	3 (16.7%)
ICI used, n(%)	Pembrolizumab	24 (82.8%)	9 (81.8%)	15 (83.3%)
	Nivolumab	1 (3.4%)	1 (9.1%)	0 (0%)
	Ipilimumab+Nivolumab	3 (10.3%)	1 (9.1%)	2 (11.1%)
	Pembrolizumab followed by Nivolumab	1 (3.4%)	0 (0%)	1 (5.6%)
KRAS, n(%)	Wild type	8 (27.6%)	5 (45.5%)	3 (16.7%)
	Mutated	9 (31.0%)	3 (27.3%)	6 (33.3%)
	Unknown	12 (41.4%)	3 (27.3%)	9 (50.0%)
NRAS, n(%)	Wild type	13 (46.4%)	6 (54.5%)	7 (41.2%)
	Mutated	0 (0%)	0 (0%)	0 (0%)
	Unknown	16 (53.6%)	5 (45.5%)	10 (58.8%)
BRAF, n(%)	Wild type	11 (37.9%)	5 (45.5%)	6 (33.3%)
	Mutated	3 (10.3%)	1 (9.1%)	2 (11.1%)
	Unknown	15 (51.7%)	5 (45.5%)	10 (55.6%)
Line of treatment of ICI, n(%)	First	21 (72.4%)	9 (81.8%)	12 (66.7%)
	Second	7 (24.1%)	2 (18.2%)	5 (27.8%)
	Third	1 (3.4%)	0 (0%)	1 (5.6%)

ICI, immune checkpoint inhibitor, KRAS, Kirsten rat sarcoma viral oncogene homolog, NRAS, Neuroblastoma RAS viral oncogene homolog, BRAF, V-Raf Murine Sarcoma Viral Oncogene Homolog B.

### Survival and treatment outcomes

**Figure 2:**
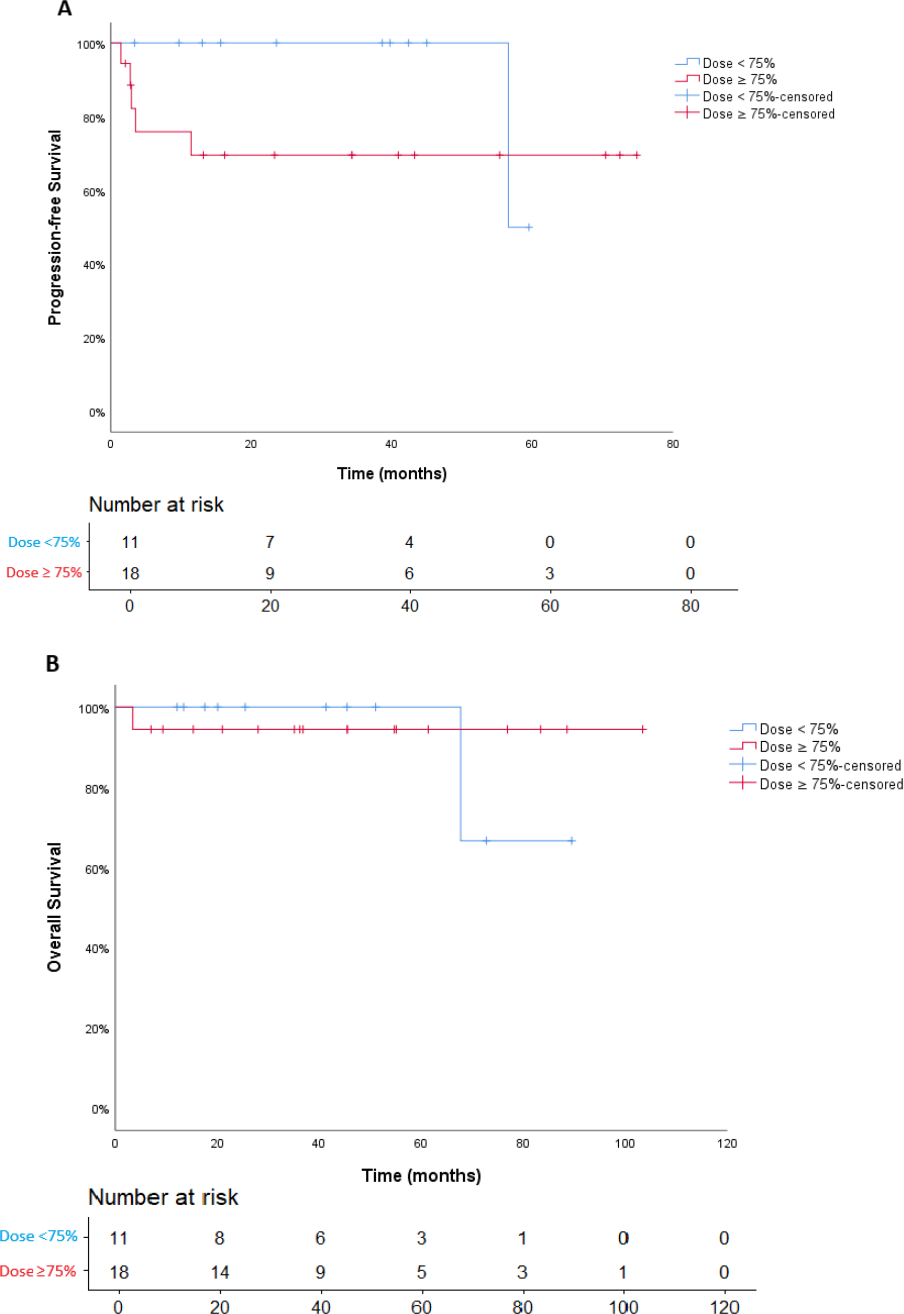
Kaplan-Meier estimates of progression-free survival **(A)** and overall survival **(B)**.

The median follow-up was 41.3 months. In the <75% dose-intensity group, the PFS at 12, 24, and 36 months was 100%, while in the ≥75% dose-intensity group, the PFS at 12 months was 69.6%, with no progression events beyond 12 months. There was no statistically significant difference in PFS between the two groups (Kaplan-Meier curve of PFS curve is shown in **Figure 2A**). The longer PFS seen in the <75% dose-intensity group may be due to the longer duration of treatment in that group. Similarly, ORR was similar between groups, with 66.7% in the <75% group and 63.6% in the ≥75% group (p=0.362), and the CBR was 72.2% and 90.9%, respectively. The estimated OS rates at 12, 24, and 36 months exceeded 90% across both dosing groups. Only one death occurred in each group and was attributed to septic shock in both cases. There was no statistically significant difference in OS between the two groups; however, the small sample size, very few events, and baseline imbalances limit the ability to draw definitive conclusions (Kaplan-Meier curve of OS curve is shown in **Figure 2B**).

**Figure 3:**
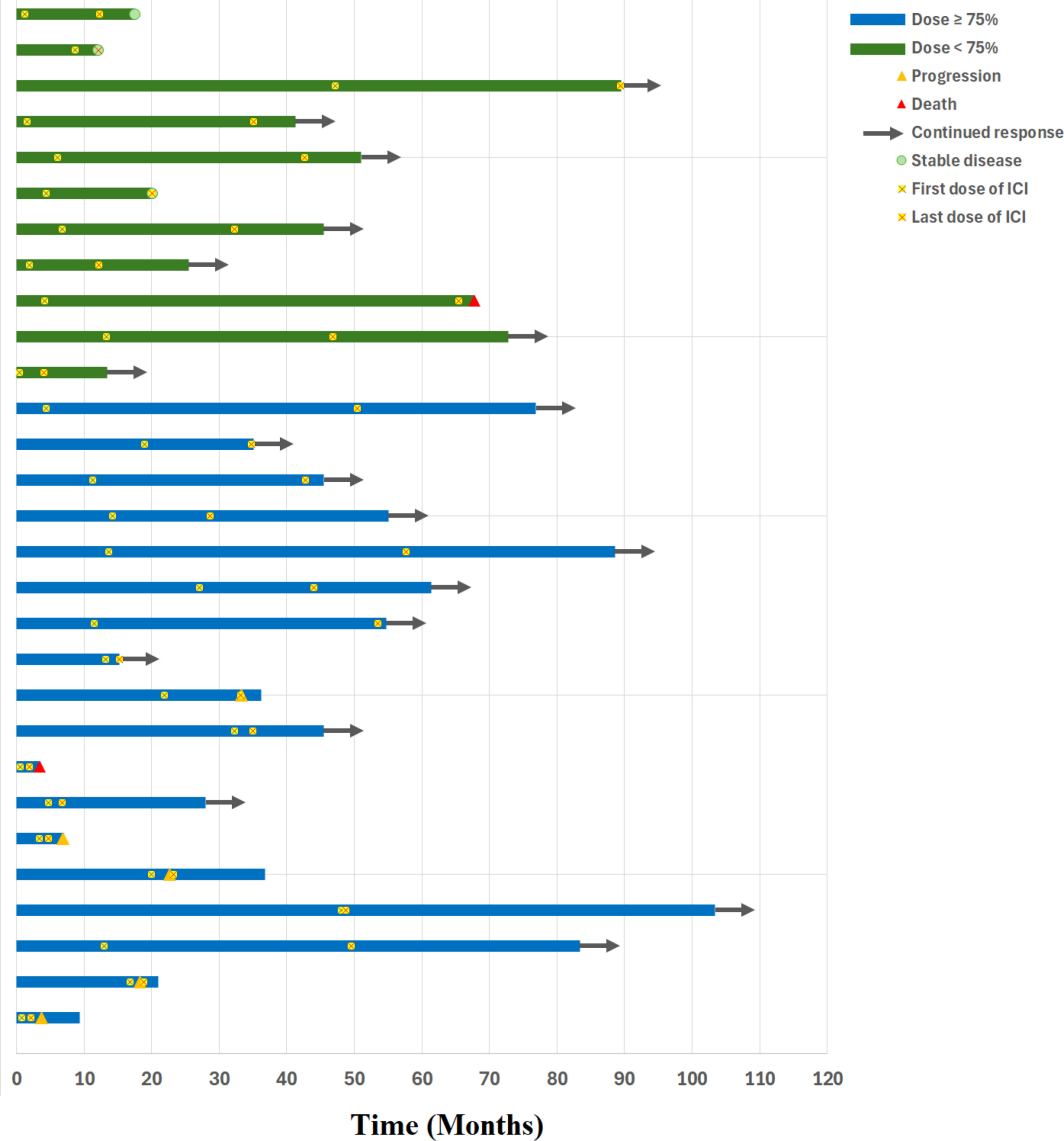
Swimmer’s plot of the overall survival.

Subgroup analysis by tumor type showed similar response rates. Among patients with MSI-H/dMMR CRC, 8 patients received <75% and 11 received ≥75%; ORR was 62.5% and 63.6%, respectively. Among patients with non-CRC MSI-H/dMMR GI cancers, 3 received <75% and 7 received ≥75%; ORR was 66.7% and 71.4%, respectively. **Figure 3** presents a swimmer’s plot illustrating OS across the entire cohort. At last follow-up, 7/11 patients in the <75% group and 12/18 patients in the ≥75% group maintained continued response to treatment.

### Treatment delivery patterns

Among the 11 patients in the <75% dose-intensity group, treatment modifications included extended dosing intervals and/or reduced doses per cycle. For the 9 patients receiving pembrolizumab in this group, the mean dosing interval was 32 days (range 24.5-38.6 days) instead of the recommended 21-day cycle, representing an approximate average of 50% interval extension. The mean administered dose per cycle was 158mg (which is approximately 80% of the standard 200mg flat dose). One patient in this group received nivolumab and he received 240mg per cycle, however, at extended intervals (the mean cycle interval was 41.5 days instead of the standard 14 days). One patient received a single cycle of ipilimumab plus nivolumab before treatment discontinuation due to surgical complication (perforated viscus); this patient maintained complete response for over 2.5 years without further therapy. In this group, patients received lower dose-intensity primarily due to financial constraints or medication shortages. The retrospective design did not allow for systematic capture of the exact reasons for reduced dose-intensity in each individual patient. The average duration of treatment was 15 months in the ≥75% dose-intensity group, compared to 25 months in the <75% dose-intensity group.

### Safety and immune-related adverse events

irAEs occurred in four patients, all of whom received ≥75% of the recommended dose-intensity. One patient experienced grade 3 erythroderma, which was managed with prednisone. Another patient developed grade 3 interstitial nephritis, requiring temporary treatment interruption and steroid therapy. A third patient had grade 3 diarrhea after two cycles of treatment, but treatment was discontinued due to disease progression. One patient developed grade 2 polyarthritis, leading to permanent discontinuation of ICI and requiring management with steroids and methotrexate. No irAEs were reported in the group of patients receiving <75% of the recommended dose-intensity.

## Discussion

Metastatic MSI-H/dMMR GI malignancies represent a minority of GI cancers (1–3). The introduction of ICIs has significantly improved outcomes compared to conventional chemotherapy (1, 5). However, one of the major barriers to widespread ICI use remains their high cost, particularly in LMICs (7). Our study demonstrates that in a tertiary center operating during a period of severe health system fragility in Lebanon, patients with MSI-H/dMMR GI malignancies who received <75% of standard ICI dose-intensity achieved clinical outcomes similar to those receiving ≥75% dose-intensity. There were no statistically significant differences in OS, PFS, or ORR between the two cohorts. These findings challenge the assumption that standard dosing ICIs are necessary to achieve optimal outcomes in this patient population and suggest that reduced dosing may be a viable alternative in resource-constrained settings.

More than a billion people reside in fragile, conflict-affected settings (13). In the MENA region, ongoing conflicts and political instability have placed widespread strains on cancer research and care, with Lebanon hosting the highest per capita refugee population worldwide and facing extreme health system fragility concurrently with economic collapse, major currency devaluation, migration of skilled personnel, shortages of medications and equipment, and conflict-related damage to facilities and workforce (11, 26). This had a significant effect on cancer care and led to treatment interruptions due to acute drug shortages in approximately 30% of oncology patients during February 2022 (11). Health systems resilience encompasses three levels of capacities: absorptive (pre-existing policies supporting immediate crisis response), adaptive (deliberate adjustments in organizational structure allowing resource reallocation), and transformative (restructuring of processes based on emerging system properties) (27), however, our experience represents neither planned absorption nor intentional adaptation. Rather, dose-intensity reduction occurred as a reactive necessity imposed by medication unavailability and economic barriers.

ICIs are among the most expensive cancer therapies, with treatment costs often exceeding $100,000 annually, and causing even insured patients to face substantial out-of-pocket costs. In the US, the annual cost of immunotherapy is $244,000 (USD) which is approximately three times the median annual income of $74,580 (USD), while in LMICs such as Lebanon, the cost of a single cycle of immunotherapy can exceed the average annual income; the price of 200 mg of pembrolizumab is about $5,400 while the average annual income is about $3,477 (7, 8). Financial toxicity associated with ICI use has been documented in other malignancies, such as NSCLC, where improved survival was accompanied by increased rates of debt collection, bankruptcy, and foreclosure, particularly among financially vulnerable patients (28).

Phase 1 and phase 2 pharmacodynamic studies have shown that PD-1 receptor saturation occurs at relatively low doses of pembrolizumab (≥1 mg/kg) and nivolumab (≥0.3 mg/kg). Notably, response rates of nivolumab plateau at specific dose thresholds, which vary by cancer type. In NSCLC, the plateau occurs at 3 mg/kg, whereas in renal cell carcinoma and melanoma, it occurs at 1 mg/kg. These studies suggest that optimal ICI dosing may vary by cancer type but is likely lower than current standard regimens. Additionally, PD-L1 receptor saturation alone is not enough to predict response; other factors, such as tumor type, tumor microenvironment, drug clearance, also play a role (15, 16).

Notably, the early trials that proved the efficacy of pembrolizumab used lower doses than the currently recommended dose. For instance, in the phase II/III trial KEYNOTE-010 for patients with NSCLC with TPS ≥1%, a dose of 2mg/kg of pembrolizumab every 3 weeks was used, and in the phase I trial KEYNOTE-001 for melanoma, doses of 2mg/kg and 10mg/kg every 3 weeks were used. Despite the fact that a dose of 2mg/kg proved to be effective and even had the accelerated FDA approval for melanoma initially, the subsequent phase III trials KEYNOTE-042 for NSCLC and KEYNOTE-006 for melanoma used higher doses: 200mg flat dose and 10mg/kg every 2 weeks or every 3 weeks, respectively, and ultimately led to the FDA approval of a flat dose of 200mg (17–22). Consequently, for patients weighing less than 100 kg, the 200 mg flat dose exceeds the 2 mg/kg weight-based dose that demonstrated efficacy in the initial trials.

Similarly, other studies comparing reduced versus standard doses of nivolumab and pembrolizumab in solid tumors have not demonstrated statistically significant differences in efficacy (23, 29). In parallel, emerging real-world evidence supports cost-mitigation approaches including dose rounding, vial sharing, weight-based or lower-dose regimens, extended dosing intervals, and shorter treatment durations, which may preserve clinical activity while substantially reducing costs. Although these strategies are largely off-label, they provide a strong rationale for prospective evaluation of optimized ICI dosing as a pragmatic and equity-oriented approach in resource-constrained settings (30). Data specific to lower doses of ICI in MSI-H/dMMR GI cancers remain limited. One retrospective study in metastatic MSI-H/dMMR CRC reported comparable outcomes with lower doses of pembrolizumab or nivolumab, though no direct comparison was made, and non-CRC MSI-H/dMMR GI cancers were not included (24).

Regarding the duration of treatment, emerging data suggests that patients may not need to continue treatment until unacceptable toxicity occurs as typically performed in clinical trials of ICIs. A systematic review and meta-analysis of 20 studies involving 1,896 patients with metastatic melanoma with median duration of treatment ranging from 3 to 34.3 months, found that most individuals who discontinued ICIs maintained durable disease control, with pooled 1- and 3-year PFS rates of 86% and 71%, respectively, and OS rates of 96% and 86% after treatment cessation. Elective discontinuation was associated with better outcomes than cessation due to toxicity (1-year PFS: 91% vs. 79%), and complete responders had the most favorable outcomes. However, longer treatment with ICIs was associated with better PFS rates (31). Novel biomarkers such as circulating tumor DNA (ctDNA) can help to guide the duration of treatment with ICIs and the timing of their discontinuation. In a study of melanoma patients treated with ICIs for a median of 11.8 months, 38 underwent ctDNA testing at the point of therapy cessation. Detection of ctDNA at discontinuation was significantly linked to subsequent disease progression (p = 0.012) (32). In another study, 34 patients with stage IV melanoma who received 1–4 cycles of ipilimumab and nivolumab and discontinued therapy due to toxicity, underwent ctDNA testing at the time of treatment cessation. For patients with detectable ctDNA, the median PFS was 6.5 months, while it was not reached (NR) for those with undetectable ctDNA (hazard ratio [HR] 4, p = 0.0023). Similarly, median overall survival (OS) was 19.4 months for patients with detectable ctDNA versus NR for those with undetectable ctDNA (HR 3.9, p = 0.024) (33). In our study, patients receiving lower dose-intensity were able to receive longer duration of treatment as compared to patients with high dose-intensity. This indicates that in resource-constrained settings, reduced ICI dosing may represent a key therapeutic strategy to maintain treatment continuity and to extend treatment duration.

Our study did not find any difference in outcomes between CRC and non-CRC MSI-H/dMMR GI malignancies treated with either standard or reduced ICI dosing. Furthermore, several patients maintained durable responses for months and even years, after discontinuing treatment. The observed results reflect the feasibility of adaptation of lower dose-intensity of ICIs in the setting of constrained access, supply chain instability and patient affordability issues.

In our cohort, irAEs were infrequent, and were observed only in the ≥75% dose-intensity group. Although interpretation is limited by the small sample size and retrospective design, this finding is consistent with evidence suggesting that toxicity risk may increase with greater cumulative exposure to immune checkpoint inhibitors. Real-world evidence from one study demonstrated that irAEs occurred in 55.9% of patients and were independently associated with longer treatment duration and greater treatment exposure, highlighting the time-dependent relationship between immunotherapy exposure and toxicity (34). These observations raise the possibility that reduced dose-intensity or treatment interruptions may contribute to a lower observed toxicity burden, although definitive conclusions cannot be drawn without prospective evaluation.

Several clinical trials have shown that lower doses of ICIs have comparable efficacy to standard dosing (24, 29). In our study, dose-intensity reduction of ICI can be viewed as a pragmatic adaptation in extreme resource-constrained settings, rather than as evidence of standard-equivalent efficacy. The findings are hypothesis-generating and can guide future prospective studies, such as pharmacokinetic−guided or non-inferiority trials. Furthermore, our experience highlights the need for: flexible oncology treatment protocols that account for supply chain instability, integration of cancer into humanitarian emergency preparedness plans, research prioritization of low-dose regimens for high-response tumors that may serve as crisis standards.

Because dose-intensity was defined over the treatment period, it represents a time-dependent exposure and may still be subject to immortal time bias, although patients could be classified as high dose-intensity even with short treatment duration. Treatment interruptions or delays may have led patients with worse prognosis to fall into the lower dose-intensity group, potentially biasing results in favor of higher dose-intensity. Strategies such as landmark analyses and time-dependent Cox regression have been used in immune checkpoint inhibitor real-world studies to mitigate such bias (34), but were not feasible due to the small sample size and limited number of events.

Our study has several limitations. First, the small sample size, censoring, tumor type heterogeneity, age imbalance between groups and the missing molecular data, may have confounded the results. Second, its retrospective design inherently carries a risk of selection and immortal time bias and limits the ability to establish causal relationships. Although dose-intensity reductions were primarily driven by system-level constraints rather than clinical selection, residual confounding cannot be excluded. Financial barriers, insurance status, and access limitations may correlate with patient-level socioeconomic factors, health status, or treatment adherence, which themselves may influence clinical outcomes. Moreover, data on patients’ financial status and insurance coverage were unavailable, as were detailed reasons for treatment interruptions or dose reductions. Third, survival data remains immature due to limited follow-up duration. Despite these limitations, this study provides valuable real-world data on ICI dosing in a rare and clinically significant subset of GI cancers. It includes both CRC and non-CRC MSI-H/dMMR malignancies and directly compares outcomes across dose-intensity groups. These findings support the development of dose-adjusted immunotherapy protocols and inform health policy decisions and reimbursement strategies in LMICs, where cost-effective immunotherapy approaches are urgently needed.

## Conclusion

As cancer care remains underprioritized in humanitarian frameworks despite affecting over a billion people in fragile settings, evidence from adaptive strategies in conflict-affected health systems is urgently needed. Our study provides exploratory observations suggesting that lower dose-intensity of ICIs may be feasible in MSI-H/dMMR GI malignancies as a pragmatic strategy to ensure continuity of cancer treatment and avoid treatment interruptions, when standard dosing is not accessible. Larger prospective studies are needed to confirm our results.

Importantly, oncology research in conflict-affected areas should explicitly incorporate concepts of health system fragility, resilience, and adaptation, as reporting clinical outcomes without contextual analysis limits the generation of actionable knowledge for a significant subset of the world’s population living in fragile and conflict-affected settings.

## Data Availability

The raw data supporting the conclusions of this article will be made available by the authors, without undue reservation.
